# Malaria Detection Using Advanced Deep Learning Architecture

**DOI:** 10.3390/s23031501

**Published:** 2023-01-29

**Authors:** Wojciech Siłka, Michał Wieczorek, Jakub Siłka, Marcin Woźniak

**Affiliations:** 1 Faculty of Medicine, Jagiellonian University Medical College, 31-008 Kraków, Poland; 2 Faculty of Applied Mathematics, Silesian University of Technology, 44-100 Gliwice, Poland; 3 Geosolution Sp. z o.o., 02-672 Warsaw, Poland

**Keywords:** neural networks, malaria, CNN, semantic segmentation network, disease detection

## Abstract

Malaria is a life-threatening disease caused by parasites that are transmitted to humans through the bites of infected mosquitoes. The early diagnosis and treatment of malaria are crucial for reducing morbidity and mortality rates, particularly in developing countries where the disease is prevalent. In this article, we present a novel convolutional neural network (CNN) architecture for detecting malaria from blood samples with a 99.68% accuracy. Our method outperforms the existing approaches in terms of both accuracy and speed, making it a promising tool for malaria diagnosis in resource-limited settings. The CNN was trained on a large dataset of blood smears and was able to accurately classify infected and uninfected samples with high sensitivity and specificity. Additionally, we present an analysis of model performance on different subtypes of malaria and discuss the implications of our findings for the use of deep learning in infectious disease diagnosis.

## 1. Introduction

Malaria is a protozoan, acute, febrile illness that has been for years one of the leading causes of death in low-income countries and thus, still remains one of the most severe public health challenges. This illness is caused by intracellular parasites of the Plasmodium genus, which are transmitted to the people via the saliva of the female Anopheles mosquito. Even though at least 150 species of Plasmodium are known, solely a few of them can infect humans: *P. falciparum*, *P. vivax*, *P. malariae*, *P. ovale*, and, still considered as a zoonotic malaria, *P. knowlesi*. Furthermore, some cases were recently reported of zoonotic infections by *P. cynomolgi* and *P. simum*; however, their global burden and clinical impact are yet to be investigated [1,2].

While *P. falciparum* accounts for the vast majority of cases and deaths, *P. vivax* is the most widespread of the malaria species [3] and poses a significant health threat, particularly in areas with intense transmission, such as Papua New Guinea. *P. ovale* and *P. malariae* are thought to be the most benign, yet the latter was associated with the lowest concentration of hemoglobin in certain regions [4] and may be associated with a high risk of anemia and subsequent hospitalization [5]. *P. knowlesi*, although rare, remains a leading cause of malaria in tropical regions of Southeast Asia with a clinical course that may resemble falciparum malaria [6].

Since *P. falciparum* is the deadliest one, most eradication efforts, including diagnostics availability and treatment efficiency, were directed toward this species. However, in order to meet WHO global targets, i.e., reducing malaria mortality rates and case incidence by 90% by 2030 along with completely eradicating malaria worldwide by 2040, it is necessary to make indispensable arrangements toward fighting off all species altogether. This may turn out to be a significant task in the light of growing drug resistance, reports of local vulnerability for malaria resurgence, instances of malaria rebounding, and diagnostic tests of a poor quality in many areas [7,8,9,10]. The latter can be improved by the introduction of new diagnostic methods, including approaches based on artificial intelligence (AI).

When addressing the medical diagnosis field, AI-based systems can be beneficial in their ability to mimic human brain in more simple tasks and create novel solutions to more complicated ones. As an example can be given solving a Rubik’s cube using simulated data and artificial neural network architecture [11] or an even more human task such as playing a Go board game [12]. By providing valid input data, the computer could find its own way of solving an advanced task and transform raw numerical data into an algorithm. As computational power grows, there is also a rising trend in applying computer vision models into the problems requiring visual classification and analysis. By introducing convolutional layers, the input matrix is compressed into vectors in a smart way, allowing for a better understanding of the image. Such networks can not only be relatively rapidly trained but evaluation can also be performed in real time by modern hardware. Such architectures can perform multiple tasks; however, the most common is image classification and labeling based on the subject of the image [13]. With more computational power came the ability to perform multi-label classification based on rectangular masking. At first, this was limited to a slow processing of single frames; however, more research scientists discovered more advanced methods, allowing for real-time evaluation [14,15]. In later years, a new branch opened the possibility of reversing the convolution process, which leads to an image as an output. Such combination is still under heavy development as its full abilities are not yet discovered; however, some of studies focused on aspects such as image compression, where the usage of encoder, decoder, and a bypass from the hidden layers allows for the network to learn pixel relations and thus compress them into fewer vectors and later decode them into the original image with very little or no visual differences. Another example can be a generative adversarial network (GAN), where generator tries to generate an artificial image from a random noise and label and a discriminator validates its trials to give valid feedback about the accuracy of the model [16]. The image generation ability allows for image upsampling by generating new, synthetic details from low resolution data by using its trained knowledge about the context of the photo and pixel relations [17]. By compressing the image, some researchers also found a way for image denoising both in computer-generated imagery and photos with results far exceeding classical denoising algorithms both in terms of evaluation times and quality [18,19]. Such type of architecture can also be beneficial in the classification field, as the creation of a semantic segmentation convolutional neural network allowed for the segmentation of images into sparse numerical clusters with each following integer describing separate abstract class. Despite the more human-like nature of the classification process, such solution also has an advantage of per-pixel accuracy, while previously described methods could only select an approximate region. With that also comes an advantage in more busy areas of the image, as the network has less training noise and learns only relevant data. Due to this, such networks are highly used in scenarios requiring the highest possible accuracy and precision as well as best understanding of the surroundings such as computer vision software for autonomous vehicles, medical systems, robots, many fields of engineering, etc. [20,21,22].

Malaria is a serious and potentially life-threatening disease caused by parasites that are transmitted to people through the bites of infected mosquitoes. The early diagnosis and treatment of malaria are crucial for the effective management of the disease and can help to reduce the risk of complications and death. One way that artificial intelligence (AI) is being used to support the detection and diagnosis of malaria is through the use of a segmentation network. This is a type of neural network that is trained to analyze images of blood smears and identify the presence of malaria parasites. The segmentation network is able to accurately identify malaria with a high degree of accuracy, currently at 9.68%. This can be a valuable tool in the fight against malaria, as it can help to rapidly and accurately diagnose cases, allowing for timely treatment and help to prevent the spread of the disease. In addition, the use of a segmentation network can help to reduce the workload of healthcare professionals and improve the efficiency of the diagnostic process. By using AI to support detection and diagnosis of malaria, we can help to improve the management and control of this important global health issue.

## 2. Epidemiology

As detailed in the recent World Health Organisation (WHO) Malaria Report, there were approximately 241 million cases of malaria worldwide in 2020, whereas the estimated number of deaths stood at 627,000 [23]. Although the total number of cases remains the same as in 2000, the ongoing eradication program initiated in the early 2000s led to the malaria case incidence decreasing from 81 per 1000 population at risk in 2000 to 56 in 2019. Likewise, deaths per 100,000 population at risk declined from 30.1 to 13.8 in 2019. It is worth mentioning that those numbers have marginally risen in the year 2020, but this is owing to the COVID-19 pandemic disruption [23].

Plasmodium falciparum is mainly found in sub-Saharan Africa, constituting 99.7% of all cases there in 2020 [23], yet it has also been a significant issue in the areas of the Western Pacific and Southeast Asia, where it also accounts for a significant percentage of infections. *P. vivax* is believed to cause nearly 14 million malaria episodes each year, being thereby responsible for roughly half of all malaria cases outside the African continent [24].

Nearly half of the world population is at risk of malaria infection every year, yet only a few of the infected will develop severe malaria. However, some groups are found to be more susceptible to severe disease, those being children aged under 5 years, the elderly, pregnant women, and patients with impaired immunity, such as those with AIDS [23].

In countries considered as not endemic, all reported malaria cases are acquired in endemic countries and are referred to as “imported malaria”. Country-level data on malaria cases between 2005 and 2015 were assessed, which demonstrated that Europe carries nearly 70% of the global burden of imported malaria, followed by the United States (15%) and Australia (2.2%) [25]. It is now mostly attributed to immigrants from endemic countries and residents who often visit areas with high malaria occurrence [26].

## 3. Life Cycle

Malaria parasites’ growth and development consist in multiple stages undergoing in both the Anopheles mosquitoes and humans. When a female Anopheles mosquito feeds itself on humans, an infective form of the parasite (sporozoites) is inoculated from the saliva to the dermis. Thereafter, Plasmodia migrate in the bloodstream to invade hepatocytes, within which they asymptomatically replicate for 7–14 days, undergoing transition to the next morphological state—schizont. This is referred to as the pre-erythrocytic stage, and corresponds to the incubation period of malaria infection. Furthermore, *P. vivax* and two sympatric species of *P. ovale* are able to form hypnozoites and remain dormant in the liver from weeks to several years before causing a relapse.

In any case, Plasmodia are eventually released from the liver as merozoites, which from now on, target erythrocytes (RBCs). Within these, parasites enter the erythrocytic stage of their life cycle, which includes repeated cycles of replication, egress, and re-invasion of other uninfected RBCs. *P. vivax* is marked with the red cell preference for reticulocytes (immature RBCs), which make up a small fraction of RBCs, leading to a lower level of parasitemia. Massive bursts of erythrocytic schizonts (final stage of infected RBC) occur every 24 h for *P. knowlesi*; every 48 h for *P. falciparum*, *P. vivax*, and *P. ovale*; every 72 h for *P. malariae* and are parallel to overt clinical features referred to as malaria paroxysm.

In tandem with the ongoing erythrocytic stage, some merozoites produce sexual forms, which takes place after 1 to 14 days since the infection onset, depending on the species [27]. This way, gametocytogenesis (sexual cycle) yields gametocytes, which circulate within human blood for several days. If another female Anopheles mosquito bite appears, these sexual forms of Plasmodia may be absorbed to its mesenteron where they will undergo full gametogenesis. The gametes fuse to produce the zygote, afterwards forming an oocyst in the midgut wall. After up to 14 days, the transmission cycle ends with new sporozoites migrating to the salivary glands, from where they can be injected to the human’s bloodstream.

## 4. Clinical Features of Malaria and General Pathogenesis

The evolution of the disease and its sequelae depend heavily on the Plasmodium species, previous exposure to this parasite, patients’ comorbidities, human polymorphisms, and the quality of the conducted treatment. According to the statistics, only a fraction of patients develop a full range of clinical manifestations as the malaria course is contained by either effective treatment, functional immune system, or precocious death [28]. Indeed, only 1% of falciparum malaria progresses to severe disease [29]. However, since certain pathological processes may still covertly proceed for a long time in case of hypnozoite presence, some patients may experience long-term health consequences that may make prior statistics less accurate [30].

The severe clinical manifestation of malaria differs between children and adults. While the former typifies high frequency of cerebral malaria (CM), severe malarial anemia (SMA), and respiratory distress (RD), adults more often develop multiorgan failure and shock [31]. Other clinical features include acidosis, hypoglycemia, jaundice, repeated convulsions, bleeding, and pulmonary edema [6]. Despite complexity and a great number of factors playing a significant role in the final form of malaria, there are a few fundamental, pathological mechanisms that are linked to the systemic development of severe disease: erythrocytes (RBC) sequestration, appearing exclusively in the *P. falciparum* infections, excessive host immune response, and endothelial dysfunction [29]. Naturally, the dynamic of pathogenesis intrinsically depends on the parasitemia level.

Severe malarial anemia remains the most common manifestation of severe malaria and simultaneously contributes the most to overall death burden [29]. It can be present in all types of malaria and, similarly to CM, has multiple underlying mechanisms. Firstly, parasites hinder normal globin gene expression, resulting in ineffective erythropoiesis. Moreover, iRBC are progressively destroyed as escaping merozoites rupture iRBCs, but less intuitively, the accelerated loss of uninfected RBC is also observed [31]. Some research even suggests that the latter accounts for 90% of acute anemia resulting from a single infection [32]. SMA is also attributed to the dysregulation of RBCs macrophage-mediated clearance, as uninfected RBCs are destructed more intensively than iRBCs.

Respiratory distress, similar to SMA, develops frequently in all types of malaria, manifesting itself in tachypnea and increased breathing effort, i.e., labored breathing, low chest indrawing, and nasal flaring [29]. Coexisting acidosis develops in a two-factor way: Plasmodium spp. produce ample quantities of lactic acid reducing blood pH but at the same time, a disrupted breathing pattern intensifies the acidosis [33]. It is estimated that 125 million pregnant women are at risk of contracting malaria. During pregnancy, parasites tend to accumulate in the placental intervillous space, provoking local inflammation. As a result of impaired blood flow, nutrient transport via the placenta, and many other underlying pathomechanisms, there is an increased risk for stillbirth, low birth weight, and later malaria infection in offspring [34].

## 5. Diagnosis

In the territories where malaria transmission occurs, each case of acute fever, i.e., elevated body temperature over 37.5 °C, should be consecutively tested toward malaria, as it remains the most common cause of that symptom there. Moreover, in all countries with stable transmission, malaria should be suspected in children with palmar pallor or a hemoglobin concentration of <8 g/dL [6].

In non-endemic countries, recent travel history should be always taken into account in case of any fever of unknown origin occurrence, as well as a diagnosis of life-threatening infections has to be conducted immediately. Parasitical diagnosis should be available within the next 2 h after the patient’s admission. If that is not the case, however, additional assessments should be performed for antimalarial treatment administration. This procedure is cardinal in rapid death prevention in light of the fact that *P. falciparum* may progress to death within 24 h [23].

The most perfect diagnostic test has to enable the identification of the particular Plasmodium species with high sensitivity, quantification of the level of parasitemia, and monitoring of patients’ response to administrated treatment. Along with that, it has to be easily available, cheap, and insusceptible to human incompetence, bias, or inter-observer variability. Although no such test exists today, many innovative methods are being vigorously developed reaching very high sensitivity and specificity. The poor diagnostic capability of any test increases both false-positive and false-negative cases, which thereby leads to either overuse of antimalarial drugs or undertreatment. While the former is associated with the development of drug resistance, the second self-evidently conduces to severe consequences of untreated malaria. Hence, the proper use of accurate diagnostic tests along with its rapid development contributes foremost to the malaria eradication proprieties [35].

As of today, WHO recommends malaria confirmation with the use of the either light microscopy technique or rapid diagnostic tests, whereas the former has been a gold standard for years. Blood smears, preferably from capillary blood, should be obtained as fast as possible, enabling a first laboratory assessment to be performed within 24 h of patient presentation. If possible, thick and thin blood films should be made collectively. A thick blood smear consists of nearly 30 layers of lysed cells, allowing for a large volume of blood to be examined at once and therefore, provide high sensitivity for malaria screening as well as simple parasite density evaluation [36]. Thin blood film, however, made from a single blood drop, enables the fast evaluation of parasite morphology, and thereby, the detection of the Plasmodium species, including their cycle stage. The only exception is observed in frequently misdiagnosed *P. knowlesi*, due to its asynchronous development and high resemblance to other species on each stage. It was reported that *P. knowlesi* was misdiagnosed as a *P. malariae* in more than a half of the examined cases [37]. Nevertheless, light microscopy has high specificity, making malaria diagnosis improbable with negative blood smear acquisition [38]. However, because that might be the case for patients with immunodeficiency, the Centers for Disease Control and Prevention (CDC) recommend to re-examine another blood film every 12 to 24 h for a total of three examinations [39].

As often regions most stricken with malaria are also affected by poverty and communication exclusion, they lack well-trained personnel along with properly equipped facilities. Consequently, where it is mostly needed, diagnosis is least accurate.

In general, novel approaches may require the development of complex computational software to drive efficient diagnosis, but also treatment monitoring. In fact, that also applies to any other branch of medicine, such as cardiovascular imaging, where diverse forms of software are being constantly developed and compared in studies [40,41].

## 6. Treatment and Prevention

Preventive procedures in endemic countries pertain to both visitors and the indigenous people, especially in the areas of constant malaria transmission risk. Basic measures comprise clothes that cover most of the body, repellents, and mosquito bed nets, such as long-lasting insecticidal nets (LLINs) [42]. There is an array of commercially available repellents, including coils, candles and sprays. While the aforementioned primarily contributes to mosquito bite prevention, other interventions, such as indoor residual spraying, prevents the transmission of infection to other people. It involves coating the walls and other surfaces of a house with a residual insecticide [29,43]. Though effective prevention against malaria itself has always been an arduous task, more and more mosquito adaptations are being widely observed in response to insecticide pressure. Apart from morphological adaptations, e.g., thickening of cuticle, behavioral changes are also noted, including shifts in biting time and negative deterrence reaction [44]. When a high risk of transmission is considered, highly effective preventive drugs are easily available. They are fundamental precautions for travelers, but do not play a significant role in continuous malaria prevention. The primarily used drug remain atovaquone with proguanil, which was observed to have up to 100% protection efficiency against *P. falciparum*. Chloroquine and hydroxychloroquine may also be used with exceptions for resistance, where mefloquine becomes an alternative [6]. Ill patient management has to be conducted forthwith, pursuant to the up-to-date algorithms, as prompt treatment implementation reduces severe malaria mortality from 100% to 20% [31]. Treatment is selected with regard to the patient’s age and general condition, region of infection, parasite species, drug resistance, and clinical form of malaria. According to the 2022 WHO guidelines for malaria management [6], artemisinin or its derivatives in combination with a longer-lasting drug is recommended as a frontline therapy for most cases. For uncomplicated malaria of all species, a three-day long Artemisinin-based combination therapy (ACT), such as artesunate with mefloquine, is preferably used, although, in non-resistant malaria, oral chloroquine or hydroxychloroquine has been also successfully applied with good tolerance as per the CDC guidelines. In the case of *P. vivax* and *P. ovale*, the main treatment has to be followed with primaquine administration for radical liver hypnozoites eradication, as the vast majority of drugs do not have any effect on them. ACTs are also willingly used among children and infants since they are generally well tolerated. Apart from ACT, all patients should always be closely monitored, as even well-treated asymptomatic hyperparasitemia has a high risk of severe malaria development. If malaria occurs again in the next 28 days following first-line ACT administration, alternative ACTs known to be effective in the region are recommended to be applied [6].

Much effort has been put toward vaccine development, but the polymorphic nature of Plasmodium makes this task utterly difficult. As of today, no fully efficient vaccine exists in worldwide use; however, the most prospective malaria vaccine, RTS, S/AS01, already undergoes pilot trials in African countries for effectiveness assessment. Since partial immunoprotection was observed in prior investigations, WHO recommends to use this vaccine for the prevention of falciparum malaria in children [23]. Nevertheless, the results of those pilot trials are highly anticipated.

## 7. Proposed Solution

The detection of Malaria can be approached by using various different techniques. One of the most accurate include visual classification. In normal conditions, such examination is performed by a human doctor and consists in the manual classification of hundreds of objects per patient within previously selected frames of blood smears. Due to this, such process is extremely slow and requires full focus of the specialist for the whole time in order to reduce oversight and misclassification rate, as the differences between healthy and infected cells can be minimal.

As such examination is defined by high repeatability and a small amount of additional stimulus, it can be near impossible for doctors to maintain the peak detection performance and accuracy during the whole day. Considering some external factors such as tiredness, short deadlines, or lack of special knowledge in the field, the accuracy can be even lower.

Many CNN techniques contain rectangular masking, such as in [45]; however, in this research for better accuracy the semantic segmentation method has been used. Such architecture can provide additional data in the form of a two-dimensional matrix with element classification for the specialist. For fast and easy verification, the data can be shown next to the original image. Such semi-automatic approach, due to its persistence, can highly reduce the error rate by providing an initial diagnosis made by the system and pointing out suspicious elements, as well as improve diagnosis time by reducing the human part from the initial analysis.

Additionally, by choosing a semantic segmentation architecture over a rectangular masking convolutional neural network, classification is made with per-pixel accuracy and thus there is better clarity and accuracy improvement on images with dense overlapping objects. Some improvement in verification is also perceived as the differences between infected and healthy cells can be better distinguished.

**Figure 1:**
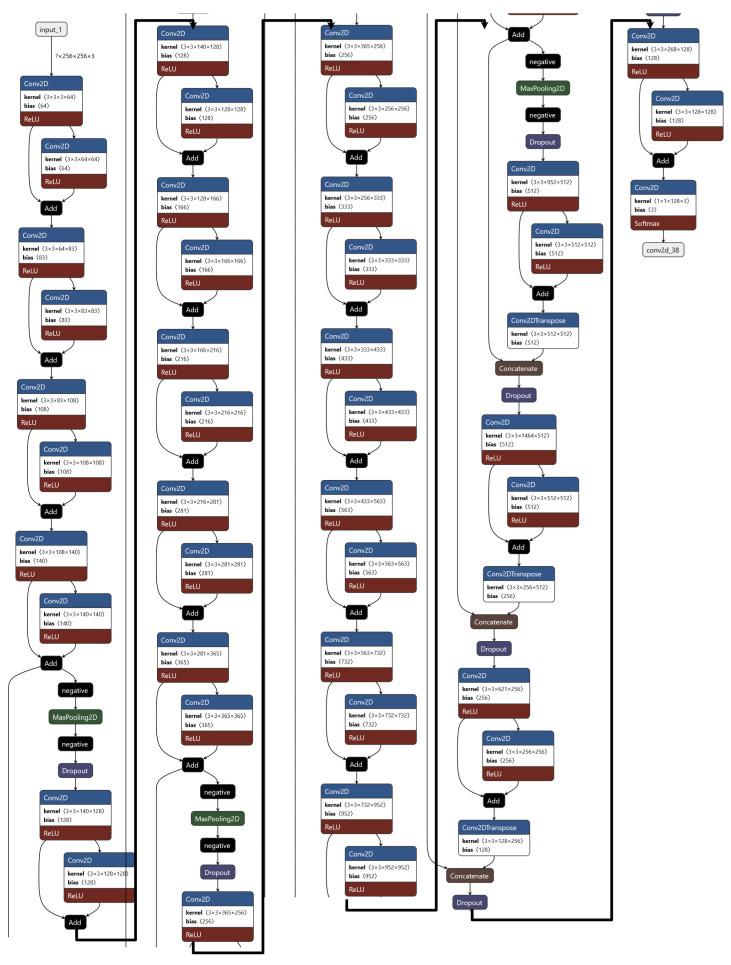
Presented deep learning model architecture is designed for the task of detecting malaria from images of blood samples. It employs an encoder/decoder scheme, with residual layers in the encoder section and U-shaped skip connections in the decoder. The encoder/decoder architecture is a common approach in image segmentation tasks, and involves two separate neural networks to process the input image and generate a detailed output segmentation map. The encoder network is responsible for extracting relevant features from the input image, while the decoder network takes these features and uses them to generate the final output. The inclusion of residual layers and skip connections can help improve the model’s performance by allowing it to more easily learn complex relationships within the data and make more accurate predictions. Residual layers are a type of layer that allows the model to learn the residual mapping between the input and output, rather than trying to learn the mapping from scratch. This can make it easier for the model to learn from the data and can improve its performance. Skip connections, on the other hand, allow for the model to directly incorporate information from earlier layers in the network into the final prediction, which can also help to improve performance. The combination of an encoder/decoder architecture with residual layers and skip connections allows the model to perform precise segmentation of the task and produce accurate results. This makes it a promising tool for detecting malaria from images of blood samples.

In this research, a novel deep convolutional neural network solution is presented. The proposed model is based on the semantic segmentation neural network idea with custom architecture and layers layout. The final developed model can be seen in Figure 1. The input of the network consists of a 300 × 300 image taken with a light microscope. Later, the encoder section downsamples the image four times using max pooling layers with a factor of 2. Such decision has been made as it allowed for better separation of cells in the compressed image. During that process, the signal is additionally enhanced by skipped connections combined using added layers and a small amount of dropout after pooling to address the issue of overfitting. All values and rates have been chosen empirically. The bottleneck section is minimal with only three layers as the experiments showed no visible gains with more complications; however, adding more layers significantly increased training and evaluation times. Finally, the decoder section consists of a mirrored encoder with skipped connections inspired by the U-Net-shaped architecture with a total number of parameters of 95,354,474. The entire model used the rectified linear unit (ReLU) Equation (1) as a main activation function and the final layer used the Softmax Equation (2). The training has been optimized with the NAdam Algorithm 1 with a constant learning rate of 0.00012.

(1) Relu(z)=max(0,z),

(2) $$\sigma \left(z_{i}\right)=\frac{e^{z_{i}}}{\sum_{j=1}^{K}e^{z_{j}}}\;\;\;for\;i=1,2,\cdots ,K.$$

**Algorithm 1** NAdam training algorithm.
1:Generate random weights,2:**while** *global error value* ε<error_value **do**3:   Shuffle the training dataset,4:   **for** each batch inside training dataset **do**5:     Compute gradient vector g on the batch,6:     Update vector *p* Equation (3),7:     Update vector *u* Equation (4),8:     Rescale vector $\hat{p}$ Equation (5),9:     Rescale vector $\hat{u}$ Equation (6),10:     Update variable $\hat{w_{s}}$ Equation (7).11:     Step = Step + 1,12:   **end for**13:   Calculate *global error* ε,14:
**end while**



### NAdam Algorithm

To improve training performance in terms of validation accuracy and training times, the NAdam training algorithm has been used. The formula can be described as follows:

(3) $$p_{s}=\beta_{1}p_{s-1}+\left(1-\beta_{1}\right)g_{s},$$

(4) $$u_{s}=\beta_{2}u_{s-1}+\left(1-\beta_{2}\right)g_{s}^{2},$$

where β parameters are constant hyper-parameters and *g* is the current gradient value of an error function. Values $p_{s}$ and $u_{s}$ are used later for computing the correlations marked as ${\hat{p}}_{s}$ and ${\hat{u}}_{s}$ according to below equations:

(5) $${\hat{p}}_{s}=\left(1-\beta_{1}\right)g_{s}+\beta_{1_{{}_{s+1}}}p_{s}$$

(6) $${\hat{u}}_{s}=\frac{u_{s}}{1-\beta_{2}^{s}}.$$

Finally, using previously calculated variables, the final formula can be defined as:

(7) $$w_{s}=w_{s-1}-LR\frac{{\hat{p}}_{s}}{\sqrt{\beta_{2_{{}_{s}}}}+\epsilon }$$

where ϵ is a small, constant value and LR is a learning rate.

## 8. The Dataset

**Figure 2:**
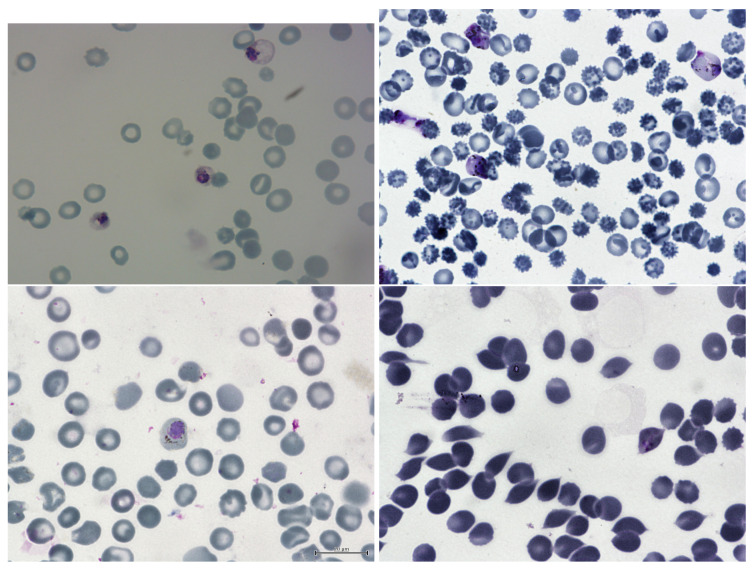
This sample dataset consists of blood smear samples that have been analyzed under a microscope. The samples contain a mixture of uninfected and malaria-infected red blood cells (RBCs). The uninfected RBCs are visible as monochromatic oval shapes with a light center and smooth surface. They have a normal appearance and do not exhibit any signs of infection. On the other hand, the RBCs that are infected with malaria appear rather different. They are shape-distorted and have a pinkish-purple color due to the presence of the malaria parasites within them. These parasites can take various forms, such as single rings or multiple eosinophilic dots, which can be seen inside the RBCs. The infected RBCs are generally larger and more irregular in shape compared to the uninfected RBCs. In addition to the RBCs, the blood smear samples may also contain singular white blood cells (WBCs). These cells are part of the body’s immune system and help to defend against infections. The WBCs in these samples are generally bigger in size and have a smooth shape with eosinophilic nuclei. They may be present in higher numbers due to the body’s immune response to the malaria infection.

The dataset, found on kaggle.com and called “Malaria Bounding Boxes”, consists of three sets of images (1364 in total) with description of around 80,000 cells with different researchers having prepared each one: from Brazil (Stefanie Lopes), from Southeast Asia (Benoit Malleret), and time course (Gabriel Rangel). Blood smears were stained with Giemsa reagent. Blood is obtained from the arm using a syringe [46] with standard procedure. Sample dataset images can be found in Figure 2.

### 8.1. Labels

In terms of labels, there are two classes of uninfected cells (RBCs and leukocytes) and four classes of infected cells (gametocytes, rings, trophozoites, and schizonts). Annotators were allowed to mark some cells as difficult if they were not clearly in one of the cell classes. The data had a heavy imbalance toward uninfected RBCs versus uninfected leukocytes and infected cells, making up over 95% of all cells.

For this research case, the above labels were simplified into two classes:

- Healthy cells;
- Infected cells.

The cells marked as difficult were attached to the “infected cells” label, as the system should mark any suspicious cells regardless its certainty and give the information to the specialist who will later inspect and classify the cell manually for double cross-validation.

A class label and set of bounding box coordinates were given for each cell. For all datasets, infected cells were given a class label by Stefanie Lopes, malaria researcher at the Dr. Heitor Vieira Dourado Tropical Medicine Foundation hospital, indicating stage of development or marked as difficult.

### 8.2. Preprocessing

**Figure 3:**
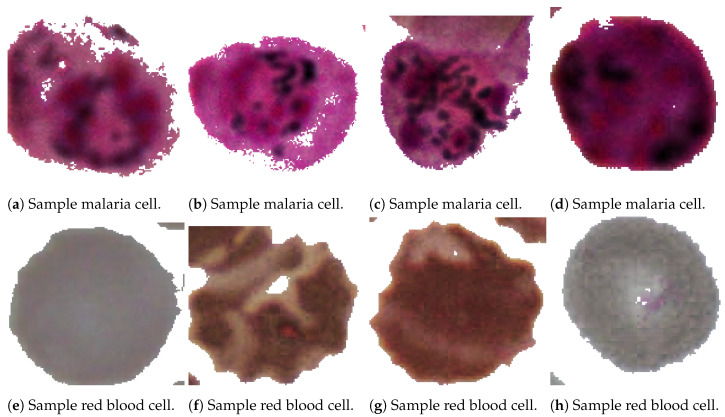
The figure presents a dataset of samples that have undergone segmentation and pre-processing. These samples have been labeled according to whether they contain malaria-infected or healthy cells. Some of the samples show clear visual differences between the infected and healthy cells, with the infected cells displaying distinct features such as shape distortion and the presence of parasites. However, most of the samples in the dataset show only subtle differences, making it difficult to diagnose the infection based on visual inspection alone. The figure provides an ideal example of the visual differences between infected and healthy cells, but in reality, most of the samples in the dataset may not be so clear-cut. This highlights the importance of using more advanced diagnostic techniques, such as molecular testing, to accurately identify and diagnose malaria infections.

As the labels are in rectangle form, which is highly inaccurate with overlapping objects and background noise, in this research the conversion to semantic segmentation mask has been made using a custom algorithm for better accuracy. The algorithm automatically clusters darker pixels inside the rectangular mask based on the pre-computed average values combined with custom heuristics and separates them from the lighter background. In later steps, some artifact removal is performed if needed. Appropriate labels are attached based on the masks’ metadata. Example separated cells are shown in Figure 3.

### 8.3. Normalization

As all images were in 8 bit, the normalization after float conversion has been made by dividing all values by 255.0.

### 8.4. Augmentation

Image augmentation can be useful when working with small datasets. Such technique is widely used in computer vision field. From simple transforms to more advanced principal component resampling [47], it can artificially enlarge the training dataset and thus improve the training process and final accuracy. In this research, for simplicity, the basic augmentation has been used, such as:

- Horizontal flip;
- Vertical flip;
- Random noise addition;
- Random rotation;
- Random zoom;
- Random hue shift.

All the masks were augmented accordingly. From the initial tests, the augmentation proved to enhance the validation accuracy by over 7%.

## 9. Our System

**Figure 4:**
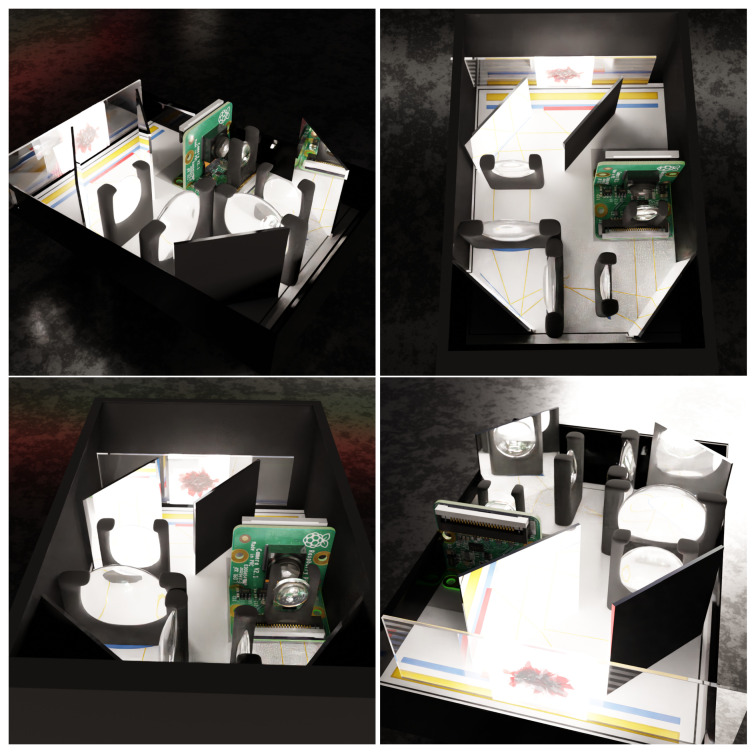
The images above show the generated images of the developed device. A micro-microscope, a type of microscope that is designed to be small and portable, is easy to use in resource-limited settings or in the field. It consists of a set of mirrors and lenses that are arranged in such a way so as to allow for the size of the lens to be reduced, while still providing a high level of magnification. This enables the microscope to be used to examine small samples, such as blood smears, for the presence of malaria parasites.

In Figure 4, we can see the image of the device we have generated. Its assumption is very simple. The sample is smeared on the glassware, and then it is illuminated from behind. The resulting image is projected by means of mirrors through lenses so that it can be properly focused. The resulting approximation is then used by the sensor that collects the results. Finally, the image is analyzed using a neural network.

**Figure 5:**
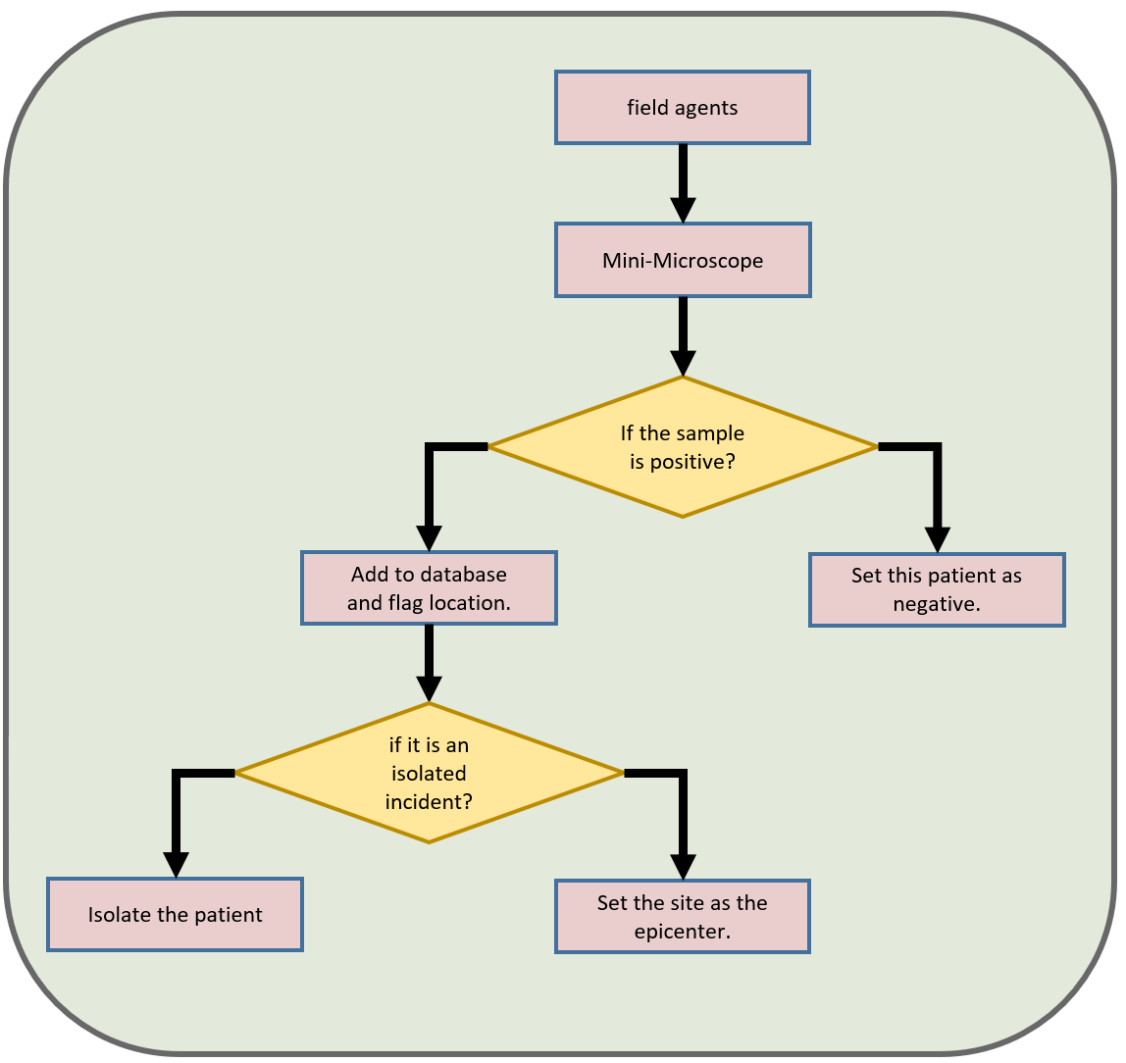
The described process is a system for detecting and responding to cases of malaria in a given population. It begins with field agents collecting blood samples from individuals and bringing them to a mini-microscope for analysis. The mini-microscope is a device that is capable of rapidly and accurately detecting the presence of malaria parasites in a blood sample. If the samples are found to be negative for malaria, they are labeled as such and the process is complete. However, if samples test positive for the disease, the process branches off and a decision is made based on the number of positive cases. If there is a large number of positive cases, the location is marked as an outbreak center. This indicates that there is a high prevalence of malaria in the area and that further action, such as increased efforts to control the spread of the disease, may be necessary. On the other hand, if there is just a single positive case, the person is isolated to prevent any further spread of the disease. This is particularly important in the early stages of an outbreak, as swift action can help to prevent the disease from spreading further. Overall, this system is designed to provide a fast and accurate means of detecting and responding to cases of malaria, with the goal of reducing morbidity and mortality rates associated with the disease.

Our system in Figure 5 of operation assumes that a field agent will test a sample taken from the patient with our device. Depending on the result, we describe the patient as positive or negative; if the patient is marked positive, their location is recorded. If there is a significant number of cases in a given location, we know that we are dealing with an epicenter and thus we can better use the resources intended to combat malaria.

**Figure 6:**
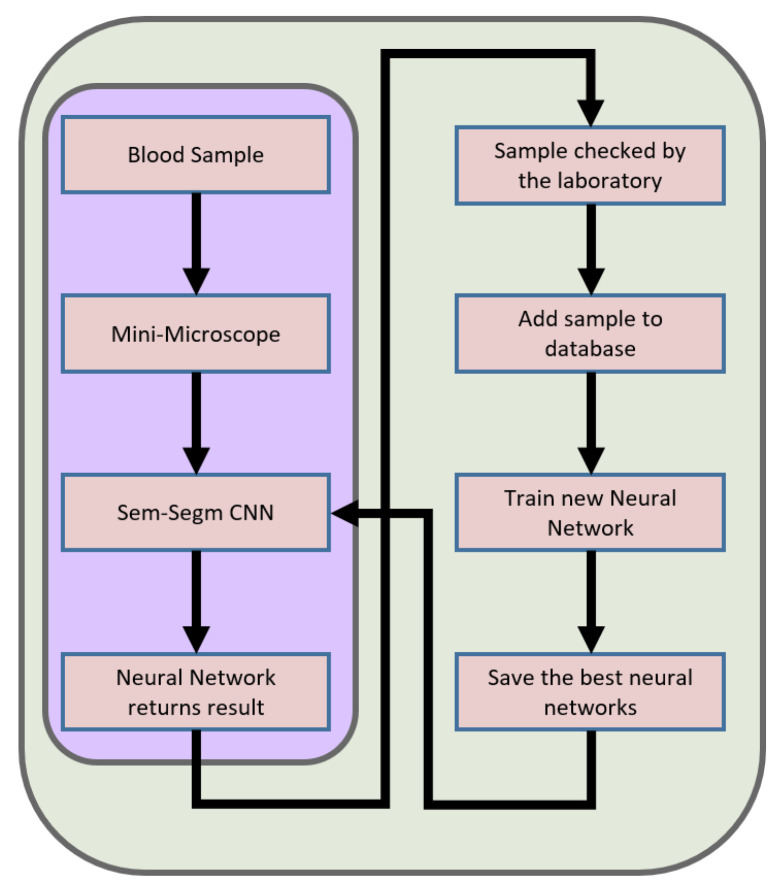
In the diagram above, we can see the data transfer in our device. When Internet access is difficult, the device switches to independent operation, which is marked in the diagram with a violet outline. If the connection is possible, the device forwards the data to enable augmentation of data used to learn the next generation of the neural network.

**Figure 7:**
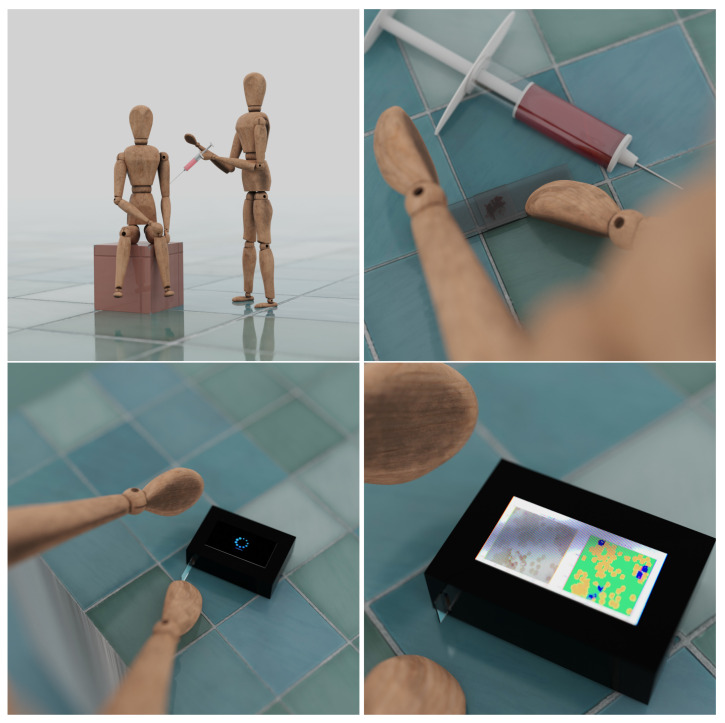
The pictures above are a graphical representations of a user use of our system. It is enough for the appropriate person to take a sample from the patient and place it on laboratory glassware, and the device will easily detect indications of malaria infection by using proposed deep learning scheme.

Figure 6 and Figure 7 show individual segments of information transmission in our system. As we assume that most of the operating time of our device will not have Internet access, we want to use every chance to supplement the database with new entries. Therefore, we start one by one with the sample that is examined by our device based on our neural network which gives as an output an estimate of the probability of abstraction classes. At this point, depending on whether the device has the ability to connect to the Internet, it sends the data, but if it works independently, the process ends. Data are sent to be analyzed by specialists and then labeled. The tagged data are sent to the general database where we teach new generations of neural networks. Ultimately, if a network performs better than the one installed on the devices, it is released as an update.

## 10. Hardware

During this research, all computations were made on a PC with the specifications below:

- CPU: Ryzen Threadripper 2950X 16c/32t;
- RAM: 128GB;
- GPU: NVidia RTX 3090 24GB.

## 11. Results

**Figure 8:**
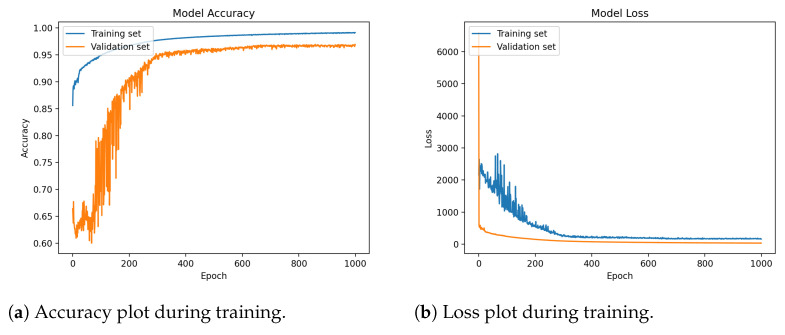
Two plots above show the performance of a deep learning model on a training and validation dataset during the training process. The plots show both accuracy and loss metrics, which are common measures of a model’s performance. The accuracy plot shows how accurately the model is able to classify the images in the training and validation datasets as it is trained. As the model is trained, the accuracy on both the training and validation datasets is expected to slowly increase, indicating that the model is learning to classify the images more accurately. Eventually, the accuracy may reach a point of overfitting, where the model is able to classify the training data with high accuracy but is no longer able to generalize well to new, unseen data. This is typically indicated by a decrease in accuracy on the validation dataset. In the end, the model reaches a per-pixel accuracy of over 99%. The loss plot shows the error of the model as it is trained. As the model learns to classify the images more accurately, the loss should decrease, indicating that the model is making fewer errors. Overall, the provided deep learning model is performing well on the training and validation datasets, steadily increasing accuracy and decreasing loss as it is trained.

The segmentation model achieves a 97.1% of per-pixel accuracy, while being light-weight enough to be rapidly run on less powerful devices during the image evaluation. On the testing hardware, the evaluation time per image is around 22 ms with the resolution of 300 × 300 but the times may vary between devices. Training plots can be seen in Figure 8. As the per-pixel accuracy is not needed in practical situations, the final accuracy has been computed using Algorithm 2 and reaches a 99.68% accuracy. The presented method creates bounding boxes on top of the segmentation mask to count all cells separately and compares results with real data provided in JSON file with the dataset. In this way, the doctor is given not only a clean mask image but also the numerical data for faster diagnosis. Comparison of the presented model with other state-of-the-art papers can be seen in Table 1.

### Visualization

**Figure 9:**
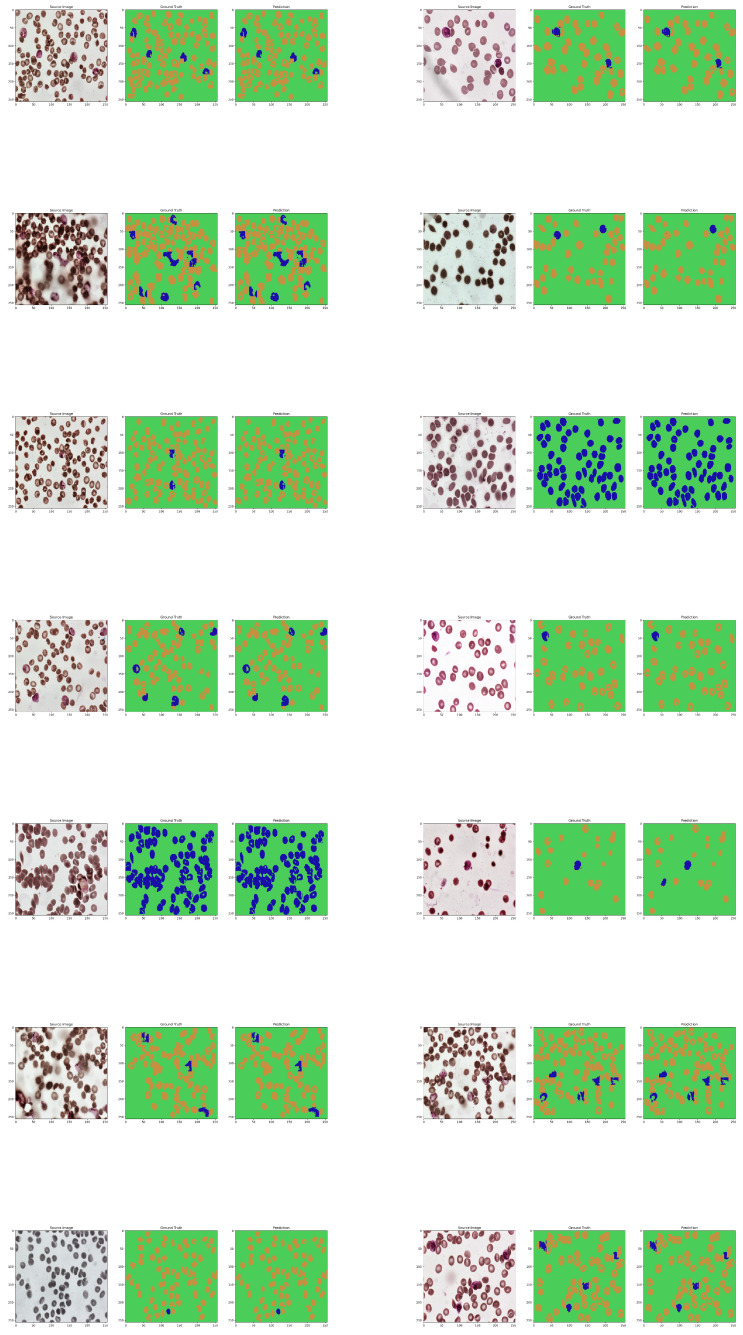
The original samples of the dataset are shown in the first row of the figure. The second and third columns show different types of masks, which are used to highlight certain features or characteristics in the images. The purpose of this evaluation is to assess the performance of the neural network in relation to the original masks. By comparing the output of the neural network with the original masks, it is possible to see how accurately the network is able to identify and classify the different types of cells in the samples. This information can be used to fine-tune the network and improve its performance for future analysis.

**Figure 10:**
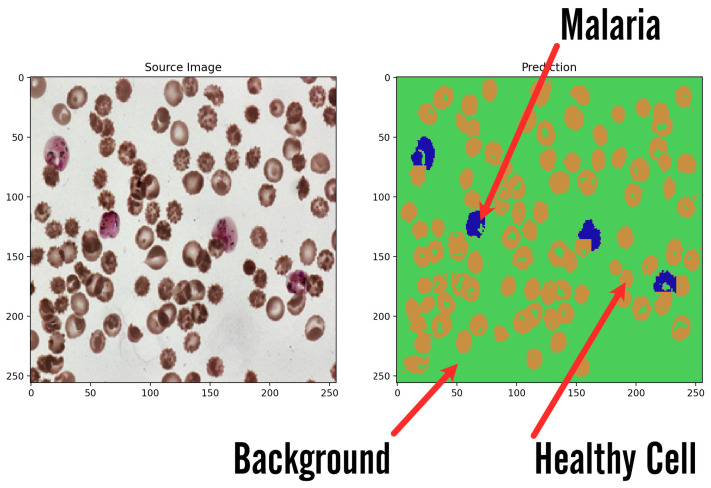
Sample result image presents how the systems returns results of classification. Input image is transformed into artificial background over which two types of blood cells are marked: healthy cells in regular features and malaria ones visible in irregular shapes and marked colors for better visibility.

The system initially outputs the data in the form of a two-dimensional mask with sparse representation of classes using integer values. Such data are optimal for being stored and analyzed by the computer; however, it presents little value for the non-technical user and requires further processing to create an informative image. Due to that, in order to make it readable, the mask has been expanded by three additional color channels and integer values from the [0,2] range and has been mapped to orange, green, and blue channels, where green is the background, orange is the healthy cells, and blue is the infected cells. Such prepared mask is presented next to the original image for fast and easy validation by the user. The results are shown in Figure 9 and Figure 10.
**Algorithm 2** Cells counting algorithm.1:Calculate Segmentation Masks,2:**for** each cell’s pixel cluster inside result mask **do**3:   cluster all neighbouring pixels with same value,4:   compute bounding box,5:   Step = Step + 1,6:**end for**7:Sum all healthy cells as β,8:Sum all infected cells as γ,9:Compute accuracy using β and γ compared to real count values from JSON file combined with the original dataset,

## 12. Conclusions

This paper presents a novel solution for rapid malaria detection using a custom semantic segmentation neural network. The model’s raw output is further processed and presented in an easy to understand and clear way, which allows for fast diagnosis and visual validation. The presented solution is able to improve the detection rate and time performance by providing additional information to the microscopy image, helping the doctor performing the evaluation to spot and analyze potential threats. After 1000 epochs of training, the network achieved a high per-pixel accuracy of 97.1% and a 99.68% accuracy for detecting a potential threat without the ideal border classification on testing data. The additional advantages of such system is high persistence even after hours of constant work, which is impossible for a human specialist, almost instant classification of the entire frame, and low cost of usage compared to the doctor.

## 13. Future Possibilities

In future works, there are many possibilities for the improvement of the presented system. Such as extension of the current dataset by additional images with adequate masks, which can rise the accuracy of the model even more, especially in more difficult situations, as well as extend the network’s knowledge about the exact shape of the infected cells regardless of the conditions. Secondly, the model’s architecture could be enhanced with more parameters fitting based on current knowledge and experience as well as future research, and thus the system could be better optimized in terms of time and detection performance. Another option is to extend the current model to all labeled abstract classes and distinguish the infected cells by the malaria development phase. Finally, more experiments could be performed with image augmentation to artificially enlarge the amount of data and possibly reduce classes imbalance for a better detection of infected cells. The use of PCR could be considered; however, more tests need to be performed.

## Figures and Tables

**Table 1:** Comparison table with other solutions.

Article	Type	Year	Accuracy
Ours	Seg-Sem CNN	2022	99.68%
Divyansh et al. [48]	CNN	2020	95.7%
Razin et al. [49]	YOLOv5 CNN	2022	96.21%
Alqudah et al. [50]	Lightweight CNN	2020	98.85%
Quan et al. [51]	ADCN	2020	97.47%
Turuk et al. [52]	Integrated CNN	2022	93.89%
Shekar et al. [53]	Fine-Tuned CNN	2020	95.99%
Rahman et al. [54]	TL-VGG16	2019	97.77%
Loh et al. [55]	Mask R-CNN	2021	94.57%
Sağlam et al. [56]	FPGA CNN	2019	94.7%

## Data Availability

We used image set BBBC041v1, available from the Broad Bioimage Benchmark Collection [Ljosa et al., Nature Methods, 2012].
